# The Nurse as an Early Warning System: A Longitudinal Surveillance Model in Pediatric Transplantation

**DOI:** 10.1111/petr.70489

**Published:** 2026-10-09

**Authors:** João Daniel de Souza Menezes, Matheus Querino da Silva, Emerson Roberto dos Santos, Mikaell Alexandre Gouvea Faria, Júlio César André, Rita de Cássia Helú Mendonça Ribeiro

**Affiliations:** ^1^ São José Do Rio Preto Medical School (FAMERP) São Paulo Brazil

**Keywords:** nursing, patient monitoring, pediatric transplantation, telemedicine

## Abstract

Posttransplant complications in pediatric patients, including rejection, infection, drug toxicity, and nonadherence, are still frequently identified at a late stage, when biomarkers have already changed and clinically meaningful harm to the graft or the patient may already be established. Current follow‐up models remain overly dependent on laboratory tests, episodic visits, and reactive responses to overt deterioration, while underrecognizing subtle clinical signals perceived by nurses and caregivers over time. This viewpoint proposes the Nurse‐as‐Sensor Framework, in which nurses act as clinical sensors capable of detecting early “soft signals,” and operationalizes it through a Longitudinal Nursing Surveillance Model, combining structured nursing consultations, remote microcontacts, a brief standardized checklist, a dynamic risk score, and graduated response pathways. We argue that this model can be implemented immediately with simple resources, increase screening sensitivity, accelerate diagnostic workup, and strengthen nursing autonomy in pediatric posttransplant care.

## 1 Introduction

Pediatric transplantation requires intensive longitudinal surveillance because children and adolescents receiving immunosuppression remain vulnerable to infection, rejection, adverse drug effects, and adherence failures, particularly during transitions from hospitalization to home and throughout ambulatory follow‐up. Yet the recognition of these events is still often triggered by abnormal laboratory findings, scheduled medical visits, or clinically obvious deterioration, preserving a predominantly reactive care model [1, 2, 3].

This limitation does not reflect a lack of monitoring, but rather a monitoring strategy that is insufficiently sensitive to the interval between formal visits and poorly structured to capture subtle changes in clinical trajectory. The literature on telehealth in transplantation shows feasibility, acceptability, and potential gains in access, adherence, and reduced acute care utilization, although pediatric evidence remains limited, heterogeneous, and often based on small or feasibility studies. Qualitative work on posttransplant care also suggests that hybrid models combining telemedicine and in‐person visits can improve continuity, access to the team, and follow‐up adherence, provided that in‐person assessment remains available when clinically needed [3, 4, 5].

At the same time, clinical experience suggests that many relevant events are preceded by nonspecific signals: behavioral change, irritability, sleep disruption, poor appetite, medication disorganization, reduced interaction, or the caregiver's perception that the child is “not quite right.” These signals are rarely systematized as formal clinical data, even though they may precede more objective physiological or laboratory abnormalities. The central hypothesis of this article is that nursing, by virtue of its longitudinal proximity to patients and families, is uniquely positioned to detect these weak early signals and convert them into actionable clinical triggers.

This model also acknowledges that stratifying surveillance intensity levels may not fully prevent delayed identification of risk factors, as changes in family dynamics or adolescent circumstances may arise unexpectedly between scheduled surveillance timepoints.

## 2 Why Are Complications Still Detected Late?

Posttransplant pediatric follow‐up remains organized around intermittent objective markers such as laboratory tests, therapeutic drug monitoring, vital signs, and scheduled face‐to‐face evaluations. This structure is necessary, but it leaves blind spots between formal contacts, precisely where behavioral changes, adherence problems, and nonspecific early manifestations emerge [4, 6, 7, 8, 9, 10, 11].

In addition, the current system often fragments information. What the caregiver observes at home, what the adolescent reports by message, what nursing staff perceive in clinic, and what appears in the laboratory are not always integrated into a single surveillance logic. The telehealth literature in transplantation indicates that interventions increasing contact and coordination with the care team may reduce logistical barriers, support adherence, and improve monitoring, but there is still no consolidated model centered on longitudinal nursing observation in pediatrics [12].

## 3 Limitations of the Current Model

Three limitations deserve emphasis. First, there is excessive dependence on biomarkers. Tests are indispensable, but they capture only part of the pathological process and often do so after clinically meaningful progression has already occurred. Second, subtle clinical signals are undervalued. In transplantation, nonspecific symptoms and changes in family routine are often treated as noise rather than potential precursors of deterioration.

Third, nursing observation is not systematically structured. Nursing staff frequently identify relevant clues during consultations, phone calls, adherence checks, and interactions with caregivers, but these clues are rarely recorded in standardized language, aggregated over time, or linked to an explicit operational response rule. Without minimal standardization, the sensitivity of care depends solely on individual experience, which limits reproducibility, training, and impact evaluation.

## 4 Conceptual Thesis: The Nurse‐As‐Sensor Framework

The Nurse‐as‐Sensor Framework (NSF) (Figure 1) proposes that nurses function as clinical sensors in pediatric transplant follow‐up. In this context, “sensor” does not mean replacing laboratory tests; it means detecting weak, contextual, and longitudinal signals that precede measurable clinical deterioration. Nursing occupies this position because it observes the patient across multiple moments, integrates biomedical and narrative data, and recognizes deviations from the child's and family's baseline.

**FIGURE 1:**
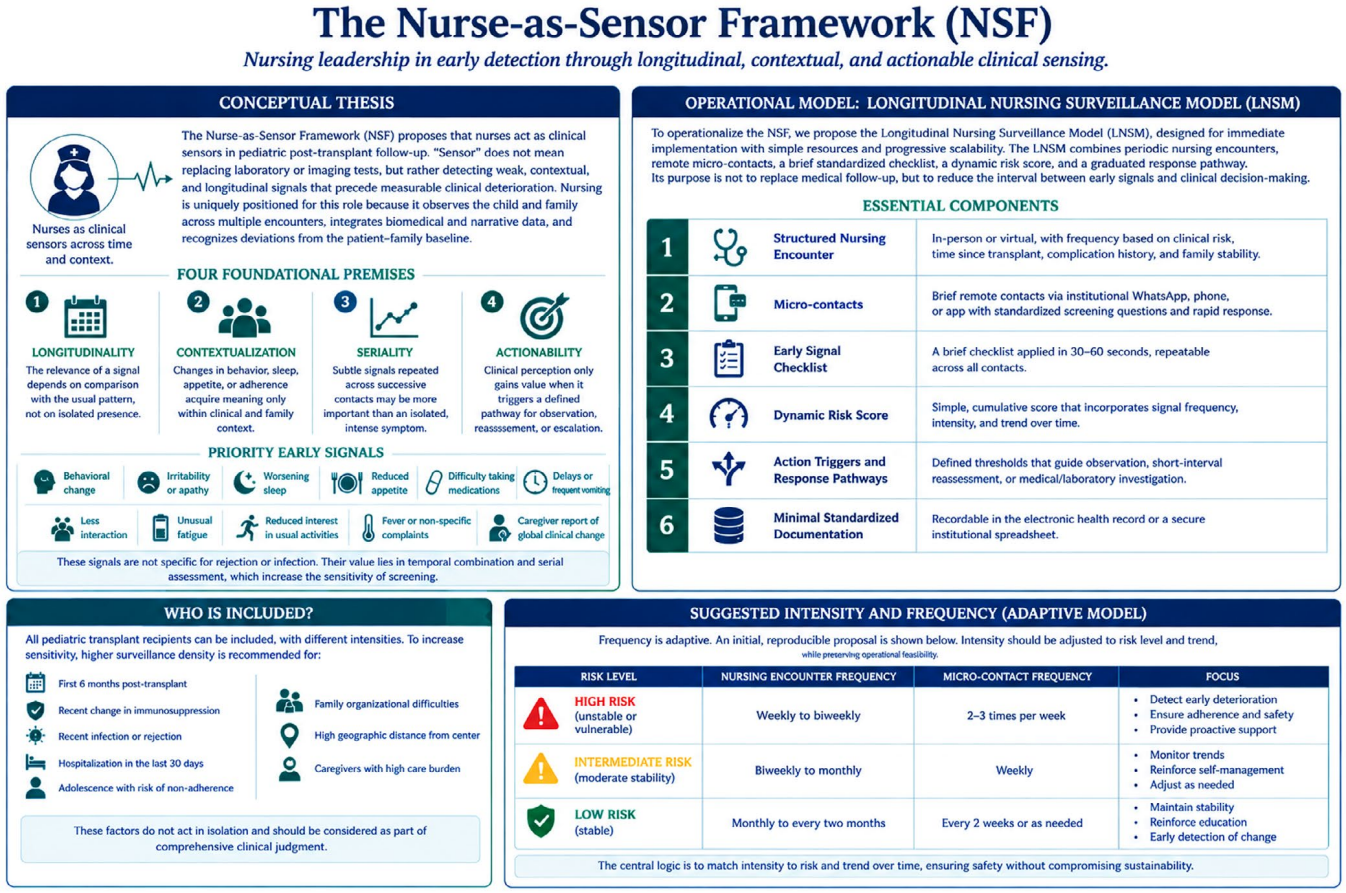
The nurse‐as‐sensor framework.

The framework rests on four premises. The first is longitudinality: the meaning of a signal depends on comparison with the usual baseline, not merely on its isolated presence. The second is contextualization: changes in mood, sleep, appetite, or adherence become meaningful when interpreted within clinical and family context. The third is seriality: discreet signals repeated across successive contacts may matter more than one isolated symptom. The fourth is actionability: clinical perception has value only when it leads to structured observation, reassessment, or escalation.

In this model, priority early signals include behavioral change, irritability or apathy, sleep disturbance, reduced appetite, difficulty taking medications, repeated delays or omissions, reduced interaction, unusual fatigue, diminished engagement in usual activities, fever or nonspecific complaints, and the caregiver's report that the child is not acting as usual. These signals are not specific to rejection or infection, but they improve screening sensitivity when interpreted as a temporal cluster.

It is important to note that this framework is designed to complement, not replace, interdisciplinary collaboration. When signals related to specific domains are detected, for example, appetite or food refusal, timely referral to a dietitian is warranted; when high family complexity is identified, engagement of social work should be pursued. The model assumes that these supports are either available within the transplant program or accessible through institutional referral pathways, with nursing serving as the coordinating hub rather than the sole responder.

## 5 Operational Model: The Longitudinal Nursing Surveillance Model

To operationalize the NSF, we propose the Longitudinal Nursing Surveillance Model (LNSM), designed for immediate implementation using simple resources and gradual expansion. The LNSM combines structured nursing consultations, remote microcontacts, a brief standardized checklist, a dynamic risk score, and graduated response pathways. Its goal is not to replace medical follow‐up but to shorten the interval between the emergence of early signs and clinical decision‐making.

## 6 Core Components

1. Structured nursing consultation, in person or virtual, with frequency determined by clinical risk, time since transplant, prior complications, and family stability.
2. Microcontacts between visits, via institutional WhatsApp, telephone, or app‐based communication, using standardized screening questions and rapid response.
3. A brief early‐signal checklist, applicable in 30–60 s and repeatable at every contact.
4. A dynamic risk score, simple, cumulative, and trend‐oriented.
5. Action pathways and triggers for observation, short‐term reassessment, or medical/laboratory evaluation.
6. Minimal structured documentation, integrable into the electronic record or a secure institutional spreadsheet.

## 7 Who Should Be Included?

All pediatric transplant recipients can be enrolled, but with different surveillance intensities. To maximize sensitivity, the highest vigilance should be reserved for the first 6 months after transplant, recent rejection or infection, recent changes in immunosuppression, hospitalization within the previous 30 days, adolescence with adherence risk, family organizational difficulties, large geographic distance from the center, and highly burdened caregivers.

## 8 Suggested Frequency

Frequency should be adaptive. An initial reproducible proposal is the following: for Level A (intensive surveillance), weekly to biweekly nursing consultations and microcontacts 2–3 times per week; for Level B (intermediate surveillance), biweekly to monthly consultations and weekly microcontacts; for Level C (maintenance surveillance), monthly to bimonthly consultations and microcontacts every 2 weeks or as needed. The central logic is to match intensity to risk and trend while preserving feasibility.

## 9 A Practical Framework That Can Be Implemented Today

The following immediate implementation framework is designed to work even without sophisticated software (Figure 2). It can be started using the medical record, a protected institutional spreadsheet, and an official messaging channel.

**FIGURE 2:**
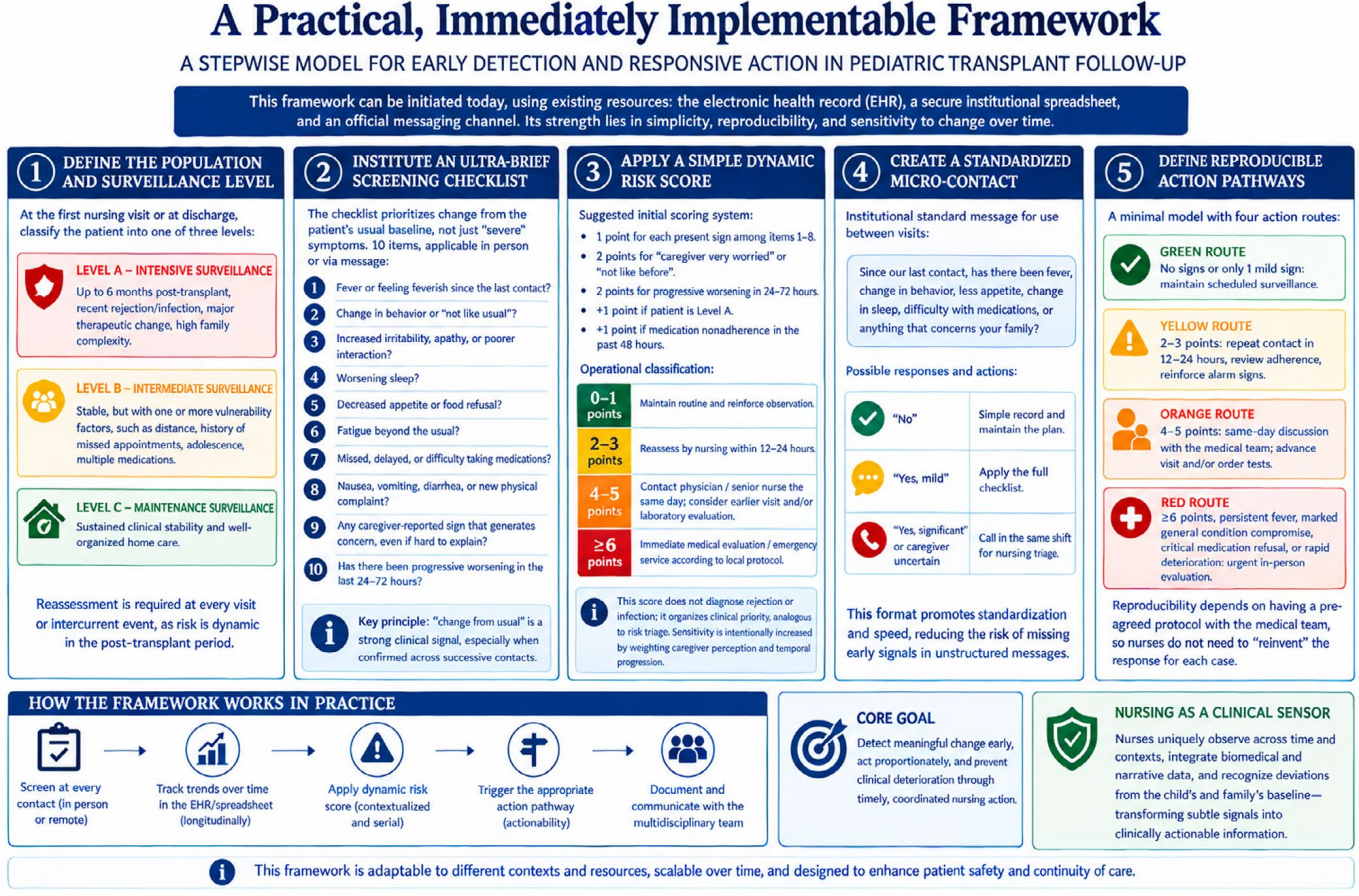
A stepwise model for early detection and responsive action in pediatric transplant follow‐up.

### 9.1 Step 1: Define the Population and Surveillance Level

At the first nursing visit or hospital discharge, classify each patient into three levels:

- Level A—Intensive surveillance: within 6 months of transplantation, recent rejection or infection, major treatment change, or high family complexity.
- Level B—Intermediate surveillance: clinically stable but with one or more vulnerability factors such as distance, missed visits, adolescence, or multiple medications.
- Level C—Maintenance surveillance: sustained clinical stability and well‐organized home care.

This classification should be revisited at every consultation or intercurrent event because posttransplant risk is dynamic.

### 9.2 Step 2: Introduce an Ultra‐Brief Screening Checklist

To make screening more sensitive, the checklist should prioritize change from baseline rather than simply the presence of a severe symptom. Suggested 10‐item tool, suitable for face‐to‐face or remote use:

1. Fever or feeling febrile since the last contact?
2. Behavior change or “not as usual”?
3. Irritability, apathy, or reduced interaction?
4. Worse sleep?
5. Reduced appetite or food refusal?
6. Unusual fatigue?
7. Missed, delayed, or difficult medication intake?
8. New nausea, vomiting, diarrhea, or body complaint?
9. Any caregiver concern that feels important even if hard to explain?
10. Progressive worsening over 24–72 h?

The key to improving sensitivity is to treat “changed from usual” as clinically strong, especially when confirmed by the caregiver across repeated contacts.

### 9.3 Step 3: Apply a Simple Dynamic Risk Score

An initial reproducible score can be structured as follows:

- 1 point for each present item among questions 1–8.
- 2 points for “caregiver very worried” or “not like before”.
- 2 points for progression over 24–72 h.
- +1 point if the patient is in Level A.
- +1 point for medication failure within the last 48 h.

Operational classification:

- 0–1 points: continue routine surveillance and reinforce observation.
- 2–3 points: nursing reassessment within 12–24 h; however, institutions should adapt this timeframe based on local staffing capacity, shift structure, and workload, recognizing that immediate 12‐h follow‐up may not be feasible in all settings.
- 4–5 points: same‐day nursing/medical discussion; consider tests.
- 6 points or more, or any severe clinical sign: immediate medical evaluation or emergency assessment according to local protocol.

This score is not intended to diagnose rejection or infection; it is designed to organize clinical priority. Sensitivity is intentionally increased by giving weight to caregiver concern and temporal progression, two domains often undercaptured in conventional follow‐up.

Shifting responsibility for initiating follow‐up from caregivers to nurses introduces important staffing and workload considerations. While some elements of the model can be automated or streamlined, the frequency of proposed interactions, required documentation, and coordination to address triggered signals will place additional demands on nursing staff who may already have limited capacity. Therefore, the long‐term feasibility and sustainability of the model depend on institutional commitment to appropriate nurse‐to‐patient ratios, protected time for surveillance activities, and ongoing evaluation of workload impact. Centers adopting this model should conduct periodic staffing‐impact assessments and adjust surveillance intensity or team composition accordingly.

The model also raises the question of how to distinguish clinically meaningful caregiver concern from persistent anxiety. Caregivers of pediatric transplant recipients often operate under chronic stress, and heightened vigilance can be adaptive or maladaptive depending on context. The LNSM intentionally gives weight to caregiver concern because qualitative evidence suggests caregivers frequently detect functional and behavioral changes before biomedical markers shift. However, repeated false‐positive signals or excessive reassurance‐seeking may warrant structured support, psychoeducation, or referral to mental health services rather than escalating medical surveillance. Nurses should be trained to recognize patterns suggesting anxiety‐driven reporting, such as frequent contacts without clear clinical change, inconsistency between caregiver reports and observed patient status, or requests for reassurance that persist after clinical reassurance, and respond with appropriate boundary‐setting, documentation, and referral. In some situations, this level of surveillance could inadvertently reinforce worry; therefore, the model should include periodic review of caregiver psychological well‐being and explicit protocols for redirecting anxiety‐driven contacts to supportive services.

### 9.4 Step 4: Create a Standardized Microcontact

Institutional standard message for between‐visit use:

Since the last contact, has there been fever, behavior change, reduced appetite, sleep change, difficulty with medications, or anything that worries the family?

Possible responses:

- “No” → simple documentation and continuation of the plan.
- “Yes, mild” → complete the full checklist.
- “Yes, significant” or unclear caregiver concern → nursing call in the same shift for triage.

This format promotes standardization and speed, reducing the risk of missing early signals in free‐text messages.

### 9.5 Step 5: Define Reproducible Action Pathways

A minimal model can include four routes:

- Green route: no signals or only 1 mild signal; continue scheduled surveillance.
- Yellow route: 2–3 points; repeat contact within 12 to 24 h, review adherence, reinforce warning signs.
- Orange route: 4–5 points; same‐day discussion with the medical team, anticipate consultation and/or tests.
- Red route: 6 points or more, persistent fever, significant compromise in general condition, critical medication refusal, or rapid worsening; urgent in‐person evaluation.

The reproducibility of the model depends on nurses not having to reinvent the response each time. The flow must be agreed upon with the medical team and documented in a simple protocol.

### 9.6 Step 6: Standardize Documentation

Recommended minimum record fields in the medical record or institutional form:

**Table:** 

Field	Suggested entry
Date/time	dd/mm/yyyy—hh:mm
Contact type	In‐person, video, phone, message
Respondent	Caregiver, adolescent, both
Present signals	Checklist 1–10
Key caregiver phrase	Brief text
Score	0–10
Trend	Better, stable, worsening
Action	Continue, reassess, discuss, escalate
Responsible staff	Name/role

Without structured documentation, there is no true longitudinal surveillance, only dispersed memory of contacts.

## 10 How to Make Screening More Sensitive

Screening sensitivity can be improved without making the system unworkable by five practical strategies.

First, replace the question “do you have any symptoms?” with “what has changed from the usual baseline?” The second formulation captures deviation and tends to reveal early signals that otherwise remain hidden. Second, explicitly weigh caregiver concern. Qualitative studies in transplantation and other pediatric chronic conditions show that caregivers often recognize functional and behavioral changes before more precise biomedical descriptions emerge.

Third, value seriality: two contacts with mild worsening may matter more than one isolated symptom. Fourth, incorporate adherence as an early clinical marker rather than merely an educational issue. Medication failure, confusion about schedules, and communication avoidance should be treated as clinical risk events. Fifth, combine brief remote triage with periodic nursing consultation. Digital monitoring alone tends to lose context and engagement over time; the literature suggests that hybrid, relationship‐centered models are more promising and more compatible with transplant care realities.

## 11 A Clinical Example

A 9‐year‐old child, 3 months after transplant and classified as Level A, responds during a microcontact that the child is “quieter,” sleeping poorly, and eating less. There is no fever. The caregiver reports that the child has not been acting normally for 2 days. Under the proposed score: behavior change (1), sleep disturbance (1), reduced appetite (1), strong caregiver concern (2), progression over 48 h (2), Level A (1) = 8 points. The indicated route is orange/red borderline, with same‐day clinical contact and consideration of in‐person evaluation and tests. In this example, no laboratory marker has yet been reported, but the model prevents the situation from being minimized as a nonspecific complaint.

## 12 Testable Predictions

The model generates empirically testable hypotheses. The first is that behavioral and routine changes precede a substantial proportion of clinically important posttransplant events in pediatric patients. The second is that structured remote microcontacts reduce the time between first signal and clinical assessment. The third is that intensive nursing surveillance reduces unplanned hospitalizations, severity at presentation, and diagnostic delays.

The fourth is that hybrid models combining consultation and structured remote monitoring outperform episodic follow‐up centered only on tests. These hypotheses can be evaluated through pragmatic pilot studies, prospective cohorts, and quality‐improvement projects. Early indicators may include time to workup after the first signal, proportion of events detected through nursing contact, unplanned hospitalization rates, follow‐up adherence, documentation completeness, and acceptability among families and staff.

## 13 Clinical and Professional Implications

This model redefines posttransplant follow‐up by shifting surveillance from already manifested events to a changing clinical trajectory. In practice, it positions nursing as a producer of clinically useful information for decision‐making rather than merely as an executor of education or a coordinator of flow. This strengthens professional autonomy and expands the responsiveness of the service without undermining multidisciplinary care.

It also integrates technology and human care in a proportionate way. The current literature cautions that telehealth should not be treated as a magic solution and that sustained benefits depend on realistic design, institutional support, and integration with usual care. For this reason, the LNSM prioritizes simple tools, common clinical language, and pre‐agreed response pathways rather than complex platforms from the outset.

## 14 Limitations and Implementation Cautions

This manuscript proposes a conceptual and operational model, not a validated score. Therefore, the thresholds should be interpreted as an initial implementation proposal subject to local refinement rather than a universal rule. Greater screening sensitivity may increase reassessment frequency and false positives, requiring a balance between clinical prudence and operational feasibility.

Moreover, microcontacts require a secure institutional communication channel, defined response times, team training, and safeguards against exacerbating digital inequities among families. Telemonitoring studies in transplantation show potential benefits, but also point to barriers related to technological access, sustained engagement, and the quality of remote assessment.

Finally, while the manuscript outlines the conceptual and operational architecture of the model, future work should explicitly address next steps for implementation and adoption. These include: (i) pilot testing in a single transplant service to refine thresholds and workflows; (ii) training packages for nursing staff on checklist administration, scoring, and differentiation of caregiver concern from anxiety; (iii) integration with existing electronic health records; (iv) family‐facing education materials explaining the purpose and limits of surveillance; (v) defined criteria for stepping surveillance up or down; and (vi) a phased evaluation plan measuring acceptability, feasibility, and preliminary effectiveness before broader dissemination.

## 15 Conclusion

Posttransplant complications in pediatric patients do not begin in the laboratory; they often begin as small changes in behavior, routine, and adherence observed in daily life. By recognizing nursing as an early warning system and operationalizing this role through a longitudinal, hybrid, and standardized model, it becomes possible to anticipate suspicion, shorten the time to evaluation, and make follow‐up more sensitive, humane, and reproducible. The proposed framework can be implemented today with simple resources and provides a concrete foundation for research, validation, and progressive implementation in pediatric transplant services.

## Funding

This research was funded by the Coordenação de Aperfeiçoamento de Pessoal de Nível Superior (CAPES), Brazil.

## Disclosure

Declaration of AI and AI‐assisted technologies in the writing process: The authors declare that no generative artificial intelligence (AI) or AI‐assisted technologies were used in the writing process of this manuscript.

## Ethics Statement

Ethical approval by a Research Ethics Committee was not required.

## Consent

This study did not involve direct data collection or human participant involvement.

## Conflicts of Interest

The authors declare no conflicts of interest.

## Data Availability

Data sharing not applicable to this article as no datasets were generated or analyzed during the current study.
